# Biodegradable Ultrasmall-in-Nano Architectures Loaded with Cisplatin Prodrug in Combination with Ionizing Radiation Induces DNA Damage and Apoptosis in Pancreatic Ductal Adenocarcinoma

**DOI:** 10.3390/cancers14123034

**Published:** 2022-06-20

**Authors:** Pei Pei Che, Ana Katrina Mapanao, Alessandro Gregori, Maria Laura Ermini, Agata Zamborlin, Mjriam Capula, Danitsja Ngadimin, Ben J. Slotman, Valerio Voliani, Peter Sminia, Elisa Giovannetti

**Affiliations:** 1 Department of Radiation Oncology, Amsterdam UMC Location Vrije Universiteit Amsterdam, Boelelaan 1117, 1081 HV Amsterdam, The Netherlands; p.che@amsterdamumc.nl (P.P.C.); danitsjangadimin@outlook.com (D.N.); bj.slotman@amsterdamumc.nl (B.J.S.); p.sminia@amsterdamumc.nl (P.S.); 2 Cancer Center Amsterdam, Department of Cancer Biology and Immunology, 1081 HV Amsterdam, The Netherlands; a.gregori@amsterdamumc.nl; 3 Center for Radiopharmaceutical Sciences, Paul Scherrer Institute, 5232 Villigen, Switzerland; ana.mapanao@psi.ch; 4 Center for Nanotechnology Innovation @NEST, Istituto Italiano di Tecnologia, Piazza San Silvestro 12, 56127 Pisa, Italy; laura.ermini@iit.it (M.L.E.); agata.zamborlin@sns.it (A.Z.); 5 Department of Medical Oncology, Amsterdam UMC Location Vrije Universiteit Amsterdam, Boelelaan 1117, 1081 HV Amsterdam, The Netherlands; m.capula@fpscience.it; 6 NEST-Scuola Normale Superiore, Piazza San Silvestro 12, 56127 Pisa, Italy; 7 Institute of Life Sciences, Sant’Anna School of Advanced Studies, 56127 Pisa, Italy; 8 Fondazione Pisana per La Scienza, Via Ferruccio Giovannini 13, 56017 San Giuliano Terme, Italy

**Keywords:** pancreatic cancer, radiosensitizing agents, chorioallantoic membrane, nanocarrier, cisplatin, gold nanoparticles, gold ultrasmall nanoparticles

## Abstract

**Simple Summary:**

The occurrence of severe normal tissue side effects and acquired drug resistance of the malignancy are the most important limitations that are associated with currently given systemic treatment in pancreatic cancer. The aim of this study was to assess the therapeutic efficacy of encapsulated cisplatin prodrug and gold nanoparticles combined with radiation to improve the toxicity profile of cisplatin and develop new multimodality treatments. Here, we demonstrated the therapeutic effect of NAs-cisPt as a platform to encourage nanomedicine in the context for future applications in multimodality treatments.

**Abstract:**

Considering the dismal survival rate, novel therapeutic strategies are warranted to improve the outcome of pancreatic ductal adenocarcinoma (PDAC). Combining nanotechnology for delivery of chemotherapeutics—preferably radiosensitizing agents—is a promising approach to enhance the therapeutic efficacy of chemoradiation. We assessed the effect of biodegradable ultrasmall-in-nano architectures (NAs) containing gold ultra-small nanoparticles (USNPs) enclosed in silica shells loaded with cisplatin prodrug (NAs-cisPt) combined with ionizing radiation (IR). The cytotoxic effects and DNA damage induction were evaluated in PDAC cell lines (MIA PaCa2, SUIT2-028) and primary culture (PDAC3) in vitro and in the chorioallantoic membrane (CAM) in ovo model. Unlike NAs, NAs-cisPt affected the cell viability in MIA PaCa2 and SUIT2-028 cells. Furthermore, NAs-cisPt showed increased γH2AX expression up to 24 h post-IR and reduced β-globin amplifications resulting in apoptosis induction at DNA and protein levels. Similarly, combined treatment of NAs-cisPt + IR in PDAC3 and SUIT2-028 CAM models showed enhanced DNA damage and apoptosis leading to tumor growth delay. Our results demonstrate an increased cytotoxic effect of NAs-cisPt, particularly through its release of the cisplatin prodrug. As cisplatin is a well-known radiosensitizer, administration of cisplatin prodrug in a controlled fashion through encapsulation is a promising new treatment approach which merits further investigation in combination with other radiosensitizing agents.

## 1. Introduction

Despite some advances obtained by regimens with combinations of conventional therapies (e.g., FOLFIRINOX and gemcitabine plus nab-paclitaxel), pancreatic ductal adenocarcinoma (PDAC) remains one of the most lethal tumors, with a five-year survival rate of only 10% [1]. Since approximately 50% of PDAC patients are diagnosed with locally advanced disease and 85–90% of patients are not eligible for surgical resection, the majority will receive some form of systemic treatment [2]. However, resistance to currently used systemic therapies as well as severe adverse events constitute main obstacles in the treatment of PDAC, prompting the development of new therapeutic strategies [3].

Cisplatin, like many other platinum compounds, has remained a staple in the systemic treatment for a variety of solid tumors, such as ovarian cancer, colorectal cancer and non-small cell lung cancer. Upon uptake in the cell, cisplatin binds to a variety of molecules, including the purine bases in DNA, ultimately causing apoptosis upon sufficient accumulation of DNA damage [4,5]. Despite successful clinical efficacy, systemic cisplatin treatment is often associated with severe adverse events, including peripheral neuropathy, ototoxicity and nephrotoxicity [6]. To attenuate its high reactivity, a substantial number of studies have synthesized cisplatin Pt(IV) derivatives with increased inertness, while exerting differential activity [7]. Another sophisticated way to approach this problem is through targeted delivery of chemotherapeutic agents by using nanotechnology [8]. The main principle relies on the application of a vehicle to increase the therapeutic efficacy through (1) improved uptake of the drug, while extenuating off-target adverse effects and (2) serve as a platform to potentiate new combination treatments within one small package.

Of particular interest within this nanotechnology field is the application of gold nanoparticles (GNPs) serving as a platform for multimodality treatments via surface modifications made on the GNP itself, acting as a vehicle [9,10]. Moreover, the high atomic number of gold make GNPs an attractive choice to enhance radiation-induced DNA damage, as reported by recent studies [11,12].

In order to overcome the resistance against cisplatin, biodegradable nanoarchitectures (NAs) were synthesized to encapsulate gold ultrasmall nanoparticles (USNPs) of approximately 3 nm in diameter together with a cisplatin prodrug as described previously [13,14]. These NAs consist of a hollow silica capsule which allow active molecules along polymer matrices with gold USNPs to reside in the inner cavity, and their rational design avoids the persistence of noble metals after the action [15]. Once taken up in the cell, the cisplatin Pt(IV) prodrug is endogenously reduced to the cisplatin Pt(II) structure via thiol-consisting proteins such as cytoplasmic glutathione (GSH).

The endogenously triggered mechanism for the drug activation reduces the intrinsic toxicity of cisplatin to off-target organs [16]. Moreover, cisPt prodrug-loaded NAs (NAs-cisPt) can be exploited for multiple actions on a single platform in combination with photothermal treatment or photoacoustic imaging applications.

While their cytotoxic effect in PDAC cells has been studied before [13], the potential of this cisplatin prodrug and gold USNPs to act as radiosensitizing agents in PDAC have not yet been explored. Therefore, we investigated the therapeutic efficacy of combining biodegradable NAs loaded with gold USNPs and the cisplatin prodrug with ionizing radiation (IR) in PDAC cells in vitro and through the development of solid tumors via the chorioallantoic membrane (CAM) model. Here, we demonstrate an increased anti-proliferative effect induced by NAs loaded with the cisplatin prodrug (NAs-cisPt), leading to increased DNA damage and consequently apoptosis, as determined via phosphorylated variant histone H2AX and increased cleaved caspase-3 levels, respectively. 

## 2. Materials and Methods

### 2.1. Chemical Reagents

The chemical reagents for the synthesis of the nano-architectures were purchased from Sigma-Aldrich and were used as received with >96% purity, unless specified otherwise. The preparation of cisplatin prodrug-modified poly(L-lysine) is described in the Supplemental File S1.

### 2.2. Cell Cultures

The MIA PaCa2 cell line was purchased from the American Type Culture Collection (ATCC^®^ 1420). The SUIT2-028 cell line was kindly gifted by Dr. Adam Frampton (Imperial College London, London, UK). These two cell lines were chosen because they were representative of the mesenchymal (MIA PaCa2) and epithelial phenotype (the SUIT2-028 is indeed a subclone of SUIT2 cells with lower metastatic ability). Primary human pancreatic ductal adenocarcinoma cells PDAC3 were isolated from a PDAC patient as described previously [17], and were chosen because of the aggressiveness of this tumor, associated with a very poor clinical outcome. All pancreatic cancer cell cultures were kept in culture in RPMI-1640 medium with L-glutamine (Gibco, #11875093, Waltham, MA, USA) supplemented with 10% heat-inactivated newborn calf serum (Biowest, Nuaillé, France) and 1% penicillin/streptomycin (BioWhittaker, Basel, Switzerland) at 37 °C and 5.0% CO2. Cells were tested monthly for mycoplasma contamination using the MycoAlert Mycoplasma Detection Kit (Westburg, Leusden, The Netherlands).

### 2.3. Nano-Architectures 

The cisplatin prodrug is an octahedral derivative of cisplatin, in which the metal center is in Pt(IV) oxidation state respect to Pt(II) in conventional cisplatin. This allows for a controlled release of cisplatin in its active form decreasing the potential systemic toxicity. Indeed, in order to be activated, the silica nanoshell of NAs-cisPt has to be eroded to expose the inner components, and then, the prodrug is reduced to cisplatin by endogenous thiol-proteins, such as GSH. The employment of the prodrug is also instrumental for the controlled integration of cisplatin within the nano-architectures. Indeed, the prodrug is covalently conjugated to the inner polymers of NAs in order to: (i) optimize the final product and homogenize the batch-by-batch features, and (ii) avoid the leakage of cisplatin from the nano-architectures before reaching the target.

Unloaded NAs and NAs-cisPt were transferred to sterile eppendorf tubes. NAs and NAs-cisPt were centrifuged to separate the ethanol for 3 min at room temperature on 14,000 rpm and subsequently the supernatant was carefully removed. Supplemented RPMI-1640 medium was added to (un)loaded NAs and sonicated for 10 s at 10% of the maximum power with a Branson high-intensity Cup Horn sonicator to obtain a homogeneous NAs solution. Treatment with cisplatin (Accord, Utrecht, The Netherlands) was prior sonicated before application to keep consistency with the other conditions.

### 2.4. Treatment with Ionizing Radiation

For combination treatment, cells were irradiated at room temperature with the 60Co-source Gammacell^®^ Research Irradiator (MDS Nordion, Ottawa, ON, Canada) at a dose rate of approximately 2.19 Gy/min 24 h after drug treatment.

### 2.5. Sulforhodamine B (SRB) Assay

Cell viability was determined via SRB assay as described previously [17]. Briefly, MIA PaCa2, SUIT2-028 and PDAC3 cells were seeded in a flat bottom 96-wells plate (VWR, Leicestershire, UK) at a cell density of 3500, 3000 and 5000 cells per well, respectively. Cells were allowed to attach for 24 h and treated with a range of different concentrations (0.25–50 μM) of sonicated unloaded NAs, NAs-cisPt, or cisplatin and subsequently fixated with TCA at a final concentration of 5.6%. After five washing steps with deionized water, cells were stained with SRB (0.4% *w*/*v* SRB in 1% acetic acid), washed four times with 1% acetic acid and air dried overnight at room temperature. Stained cells were rehydrated in 10 mM Tris base buffer. Optical density was measured on a BioTek plate reader (BioTek Instruments Inc., Winooski, VT, USA) at 490 nm. Data were obtained from three individual experiments in triplicates.

### 2.6. Colony Formation Assay

Between 500 and 10,000 cells were seeded per well in 6-well plates (VWR, Leicestershire, UK) and kept in culture under normal cell culture conditions for 5 h. After immediate attachment, cells were treated with sonicated NAs(-cisPt) and cisplatin as described before. After 7 to 10 days, colonies were washed twice with PBS, fixated in 100% ethanol (Merck, #1009832500, Darmstadt, Germany) for 15 min, stained with 10% Giemsa stain solution (Merck, Darmstadt, Germany) for 1 h and finally, cells were washed five times with deionized water. Colony formation capacity was determined by counting colonies (>50 cells) using the GelCount colony counter (Oxford Optronix, Oxford, UK), as described previously [18]. Data were obtained from at least three individual experiments in triplicates. Plating efficiency (PE) was determined by the following formula:

(1) $$\mathrm{PE}=\frac{number\;of\;treated\;colonies}{number\;of\;untreated\;colonies}$$

Subsequently, the survival fraction (SF) was calculated by the following formula:

(2) $$\mathrm{SF}=\frac{PE\;of\;treated\;colonies}{PE\;of\;untreated\;colonies}$$

### 2.7. Western Blot

To study the effect of the combination treatment, proteins from pre-treated cells with NAs, NAs-cisPt and cisplatin and/or γ-radiation were extracted at specified time points. After treatment, cells were washed twice with ice-cold PBS and lysed using RIPA Lysis Buffer System (Santa Cruz Biotechnology, sc-24948, Dallas, TX, USA) with supplied protein cocktail inhibitors, 1 mM sodium orthovanadate, 2 mM PMSF, and additionally supplemented with 1 mM β-glycerophosphate (Sigma, G6251) for 15 min on ice. Lysates were sonicated and centrifuged for 10 min at 14,000 rpm and 4 °C. The supernatant was collected and the protein concentration was estimated using the Micro BCA Protein Assay Kit (Thermo Scientific, #23235, Waltham, MA, USA). Fifty µg of each condition was loaded on pre-cast 4–15% SDS-PAGE gels (BioRad Laboratories Inc., Hercules, CA, USA) and subsequently transferred to PVDF membranes. Membranes were stained overnight at 4 °C according to manufacturer’s protocol with primary antibodies of rabbit anti-γH2AX (Cell Signaling Technology, #2577, 1:1000) and anti-β-actin (Cell Signaling Technology, #4967, 1:1000). Following incubation with secondary goat anti-rabbit HRP-conjugated antibodies (Cell Signaling Technology, #7074, 1:5000), protein expression was detected with Amersham™ ECL™ Prime™ (GE Healthcare, #RPN2232, Chicago, IL, USA). All uncut, original Western blots used to arrange the figure are found in Supplemental Figure S2.

### 2.8. Immunofluorescence

SUIT2-028 and PDAC3 cells were seeded into 8-well Lab-Tek™ II Chamber Slide Systems (Thermo Scientific, #154941, Waltham, MA, USA) and treated for 24 h with sonicated NAs-cisPt followed with 4 Gy γ-radiation. At specified time points, cells were fixated with 4% paraformaldehyde in PBS solution for 10 min and permeabilized with 0.1% Triton X100 in PBS solution for 30 min at room temperature. After 1 h of blocking in 1% BSA in PBS solution at room temperature, slides were incubated with rabbit anti-cleaved caspase-3 (5A1E), Asp175 antibody (1:500) overnight at 4 °C followed by goat anti-rabbit IgG H + L Secondary Antibody, Alexa Fluor 488 (Abcam, ab150077, Cambridge, UK, 1:1000). After one final wash in 1X PBS, slides were mounted with ProLong™ Diamond Antifade mountant with DAPI (Invitrogen, P36966, Waltham, MA, USA) and cured for 24 h at room temperature and stored at 4 °C in the dark. Slides were imaged as described, using a home-build optical microscope set-up based on an inverted Axiovert200 microscope body (Carl Zeiss, Oberkochen, Germany), a spinning disk unit (CSU-X1, Yokogawa Electric, Musashino, Tokyo, Japan), emCCD camera (iXON 897, Andor Labs, Morrisville, NC, USA). Laser settings and exposure time were adjusted with IQ software (Andor Labs).

### 2.9. Chicken Chorioallantoic Membrane (CAM) Assay

The CAM assay was performed as described previously [19]. Briefly, fertilized white leghorn chicken eggs from a local supplier (Het Anker B.V., The Netherlands) were placed horizontally and automatically rotated every 2 h on a tilting rack in a fan-assisting FIEM MG 140/200 Rurale hatching incubator (FIEM, Guanzate, Italy) at 37.8 °C and 70% air humidity. On embryonic developmental day (EDD) 3, a small opening was made on the top of the egg, sealed with tape and placed upright with no tilting in the incubator henceforth. On EDD6, the opening was made bigger and a small laceration was made on visible micro blood vessels using a paper tissue. Cells were resuspended in 50% cold growth factor-reduced Matrigel^®^ (Corning Life Sciences, #354230, Amsterdam, The Netherlands) and 10^6^ cells for both PDAC3 and SUIT2-028 were deposited into the laceration. We selected these two cells because they were representative of a more and less aggressive PDAC phenotype, respectively.

On EDD10, visible tumors were detected and 8–10 tumor-bearing eggs were randomized into different groups. Sonicated cisplatin, unloaded NAs and NAs-cisPt in 0.9% (*w*/*v*) saline solution were topically applied close to the tumor. For combination treatments, whole eggs were subsequently irradiated with 4 Gy of γ-radiation at room temperature in a ^60^Co-source Gammacell^®^ Research Irradiator 24 h post-drug treatment. Images were taken with an Optech LFZ stereo microscope (Optech, Troy, MI, USA). Tumor volumes were measured as (*length*)^2^ × *width* × 0.5 in mm. On EDD17, tumors were harvested and washed in PBS before fixation in zinc fixative (0.5 g calcium acetate, 5.0 g zinc acetate, 5.0 g zinc chloride in 1 L of 0.1 M Tris Buffer, pH 7.4) with 5.0% DMSO at 4 °C or flash frozen in liquid nitrogen for further down-stream applications.

Terminating the experiments at EDD 17 was based on an optimized experimental schedule to facilitate the handling of the models. Additionally, this ensures a humane endpoint prior to the full development of the chick embryo, in particular of the pain perception [19].

### 2.10. Immunohistochemical Staining

Slices of 3 µm CAM-derived tumors were cut from paraffin-embedded CAM specimens, which were obtained by fixing the excised CAM tumors in 4% paraformaldehyde overnight at 4 °C, and then placing them into embedding cassettes into a tissue embedding station with an increasing graded alcohol series (50%, 70%, 80%, 95% ethanol, xylene and paraffin). These sections were deparaffinized by a decreasing graded alcohol series to double-distilled water (xylene, 95%, 80%, 70%, 50% ethanol, double-distilled water) and then used for histopathological analyses with hematoxylin and eosin (H&E) and immunohistochemistry (IHC), using anti-cytokeratin 19 (Clone EP72 1:200; Epitomics, Berkshire, UK) and anti-γH2AX antibody (#9718, 1:200; Cell Signaling Technology). Visualization was obtained by BenchMark Special Stain Automation system (Ventana Medical Systems, NY, USA).

### 2.11. Quantitative Extra-Long Polymerase Chain Reaction (XL-PCR)

For quantitative XL-PCR, PDAC cells were treated with 3 µM cisplatin or NAs-cisPt or irradiated with 4 Gy 24 h post-treatment for combination treatment. After 72 h, DNA was extracted using the TRIzol Reagent (Invitrogen, #15596026) according to manufacturer’s protocol. For CAM-derived tissues, paraffin-embedded tumor samples were sectioned using the microtome and dewaxed prior to DNA extraction. XL-PCR was performed as described previously [20].

### 2.12. Inductively Coupled Plasma–Mass Spectrometry (ICP-MS)

Tumors harvested on EDD17 were dried overnight at 80 °C, until constant weight was obtained. The dried samples were transferred to pressure vessels, added with ~3 mL nitric acid, and heated up to 150 °C for 30 min. Upon acid evaporation, freshly made aqua regia (3:1 HCl: HNO3 molar ratio was added to the samples for further digestion. The digested and dried samples were then added with 3 mL of 3% nitric acid solution. The amounts of gold and/or platinum were determined after analysis on Agilent 7700 ICP-MS, using standard calibration curves.

### 2.13. Statistical Analysis

Statistical analyses were performed in GraphPad Prism version 9.1.0 (GraphPad Software, San Diego, CA, USA). One-way ANOVA test was performed for the cell viability data. Two-way ANOVA with multiple comparisons test was performed for the XL-PCR and cleaved caspase-3 data. *p*-values less than 0.05 were considered to be statistically significant.

## 3. Results

### 3.1. NAs-cisPt Inhibits Cell Proliferation in Cisplatin-Sensitive PDAC Cells

**Figure 1:**
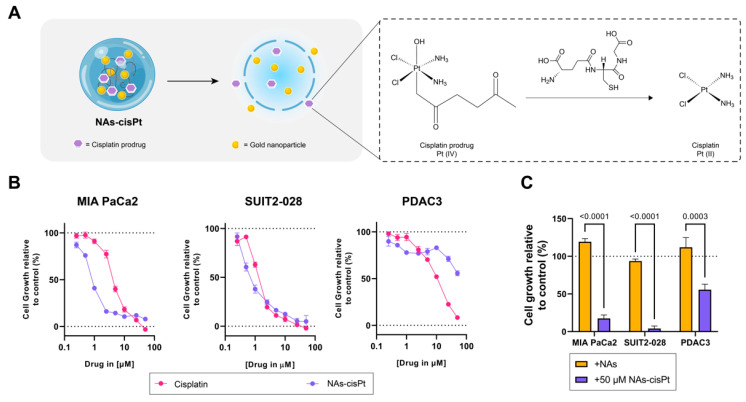
Cisplatin prodrug-loaded NAs inhibit proliferation in PDAC cells sensitive to cisplatin. (**A**) Schematic overview of NAs-cisPt. The cisplatin prodrug is loaded into the inner cavity of the nanoparticle along with gold USNPs with a diameter of 3 nm. Upon uptake by cells, the silica shell gets endogenously biodegraded to release the gold USNPs and cisplatin prodrug, which gets reduced to its active cisplatin structure. (**B**) MIA PaCa2, SUIT2-028 and PDAC3 were seeded and treated with a range of concentrations of cisplatin and NAs-cisPt for 72 h. Cell viability was assessed via SRB assay. Representative cell viability curves relative to control are shown. (**C**) Treatment comparison between NAs corresponding to the same amount of gold USNPs (2.8 ug Au total) present in 50 uM NAs-cisPt for 72 h in all three PDAC cells. Data show the mean ± S.E.M. of three individual experiments performed in triplicates.

In order to determine differential effects not only between cisplatin and the cisplatin prodrug loaded in NAs (NAs-cisPt), but also the presence of gold USNPs in NAs, we used PDAC cells of different phenotypes, such as mesenchymal-like MIA PaCa2, epithelial-like SUIT2-028 and an aggressive primary PDAC culture (PDAC3) [21]. NAs-cisPt inhibited proliferation in all three PDAC models, with MIA PaCa2 and SUIT2-028 being the most sensitive PDAC cultures (Figure 1B). Unloaded NAs corresponding to the same amount of gold USNPs (total of 2.8 µg Au) in 50 μM NAs-cisPt did not affect cell proliferation, indicating that the cytotoxic effect is derived from the release of the cisplatin prodrug (Figure 1). The IC_50_ for NAs-cisPt was calculated considering the amount of the cisplatin prodrug derived stoichiometrically from the amount of platinum within NAs-cisPt. The IC_50_ concentrations for both cisplatin and NAs-cisPt treatments for all three PDAC cultures are as reported in Table 1.

### 3.2. NAs-cisPt Effectively Inhibits Colony Formation in PDAC Cells

**Figure 2:**
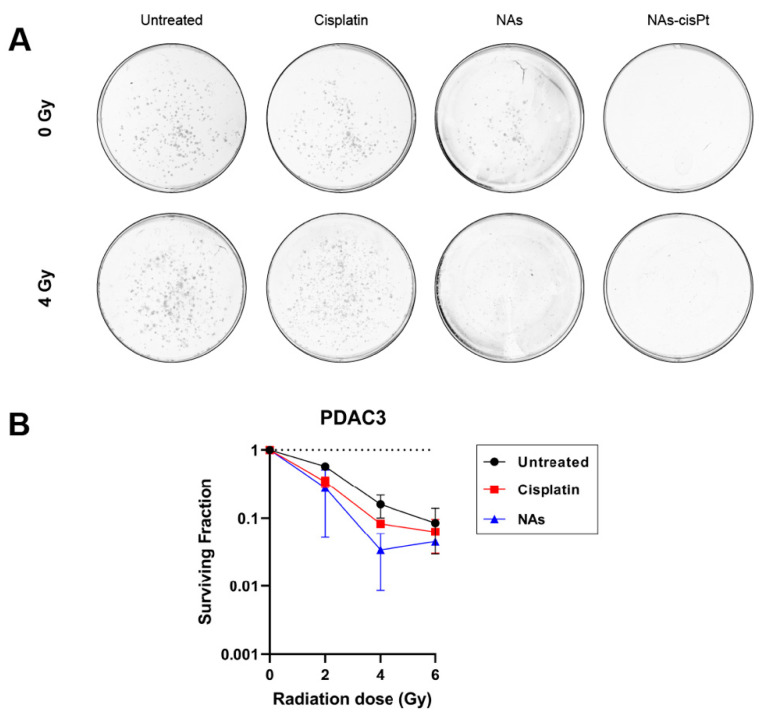
NAs-cisPt inhibits colony formation in PDAC cells. (**A**) Representative images of PDAC3 colonies without and with 4 Gy IR formed after 7–10 days of treatment with 1 μM cisplatin, NAs or 1 μM NAs-cisPt. The amount of gold USNPs in NAs (3.1 μg Au total) is equal to 1 μM of NAs-cisPt. (**B**) Cell survival curves of PDAC3 cells after treatment with cisplatin and NAs. Data show the mean ± S.D. of two individual experiments performed in triplicates.

Since cisplatin has been widely used as a radiosensitizing agent in combination with ionizing radiation, we investigated if the cisplatin prodrug or the gold USNPs could augment the radiation effect in our PDAC models. Intriguingly, increasing the amount of gold USNPs within NAs-cisPt did, however, decrease the cell viability in MIA PaCa2 cells (Supplemental Figure S1), suggesting that gold USNPs affect the proliferation in PDAC cells. To this end, we henceforth treated PDAC cells with NAs-cisPt at an increased concentration of gold USNPs (78.6 μg/mL Au) to investigate the effect of gold USNPs in combination with IR as well. Colony formation data were only obtained for PDAC3, as SUIT2-028 colonies were too diffuse to accurately visualize individual colonies. Notably, NAs-cisPt completely inhibited colony formation in PDAC3, despite equal concentration of free cisplatin (Figure 2A). Unlike NAs-cisPt, cisplatin treatment, however, did not completely abrogate the clonogenic capacity of PDAC3 cells, but did decrease the survival of PDAC3 with increasing radiation dose. Cell survival curves of PDAC3 cells treated with cisplatin and NAs were evaluated. Interestingly, NAs-treatment showed a decreasing trend in PDAC3 survival in a radiation dose-dependent manner, comparable to cisplatin treatment (Figure 2B).

### 3.3. NAs-cisPt Prolongs Induction of DNA Damage Marker γH2AX by Ionizing Radiation

To investigate the inhibited colony formation capacity after NAs-cisPt treatment in PDAC cells, we evaluated the induction of double-stranded break (DBS) formation through the expression of H2A.X at phosphorylation site Serine 139 (γH2AX) after combination with 4 Gy at different time points. The phosphorylation of H2AX plays indeed a pivotal role in the DNA damage response and is required for the assembly of DNA repair proteins at the sites containing damaged chromatin, as well as for the activation of checkpoint proteins which arrest the progression of the cell cycle. Thus, the analysis of γH2AX expression can be used to detect the genotoxic effect of different physical and chemical insults.

**Figure 3:**
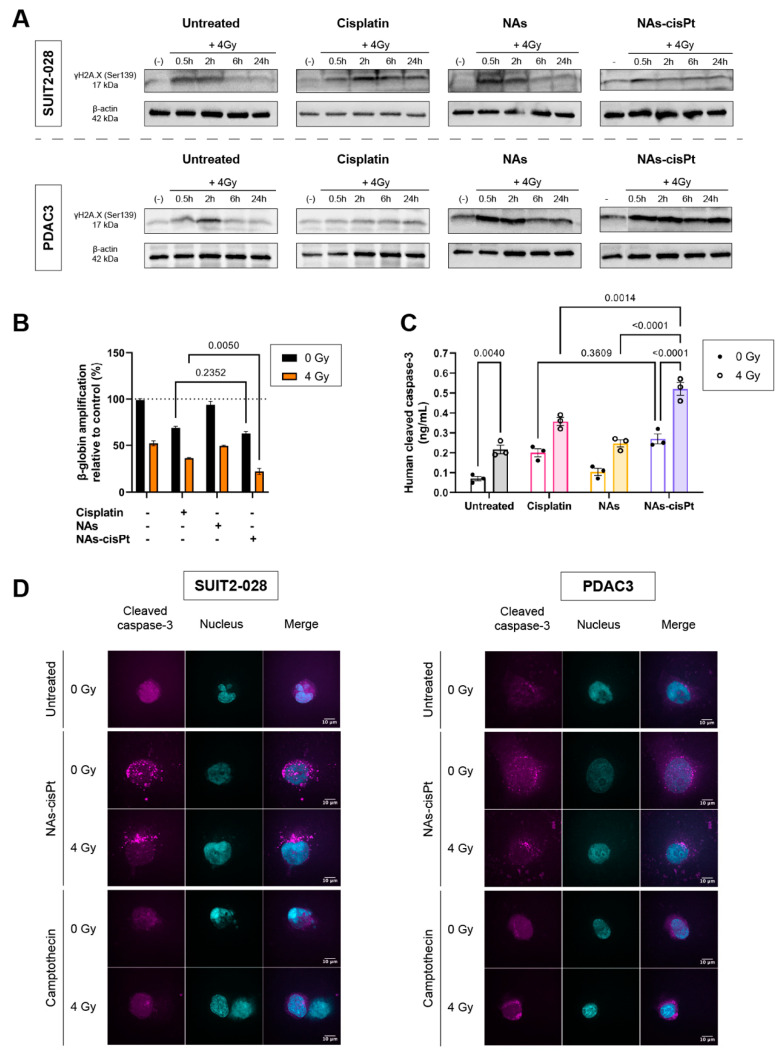
NAs-cisPt prolongs formation of DNA double-stranded breaks in PDAC cells, leading to apoptosis induction. (**A**) Protein expression γH2A.X. (17 kDa) and β-actin (42 kDa) were assessed via Western blot in SUIT2-028 and PDAC3 cells. Prior to irradiation, cells were treated for 24 h with 1 μM cisplatin or NAs(-cisPt). The gold USNPs in NAs corresponded to the same amount in 1 μM NAs-cisPt (4.72 μg Au in total). The unirradiated condition (-) was collected simultaneously with 0.5 h post-radiation. PDAC3–NAs-cisPt was re-arranged as can be seen and explained in detail in Supplemental Figure S2. (**B**) Amplifications of β-globin were assessed in SUIT2-028 after 24 h of exposure with cisplatin, NAs or NAs-cisPt with (out) 4 Gy radiation via XL-PCR. (**C**) Cleaved caspase-3 levels were measured in SUIT2-028 in ng/mL after 24 h of treatment with a commercial kit. (**D**) Representative images of immunofluorescence stainings at a magnification of 100× of cleaved caspase-3 (magenta) and nucleus (DAPI, cyan) in SUIT2-028 and PDAC3 cells after 72 h. After 24 h of incubation with 3 μM cisplatin or NAs-cisPt, cells were irradiated with 4 Gy. Camptothecin-treated cells were taken as positive control of apoptosis induction. Fluorescence intensities of cleaved caspase-3 were scaled to the same range for all conditions. Scale bar represents 10 μm. All uncut, original Western blots used to arrange the figure are found in Supplemental Figure S2.

In a similar manner to the untreated condition, γH2AX expression reverted back to the unirradiated condition in NAs-treated cells at 6 h post radiation. Contrary to the untreated group, NAs-cisPt inhibited the repair of DNA damage starting from 30 min and prolonged up to 24 h post radiation (Figure 3A and Supplemental Figure S2).

### 3.4. NAs-cisPt Augments Ionizing Radiation-Induced DNA Damage Leading to Apoptosis

**Figure 4:**
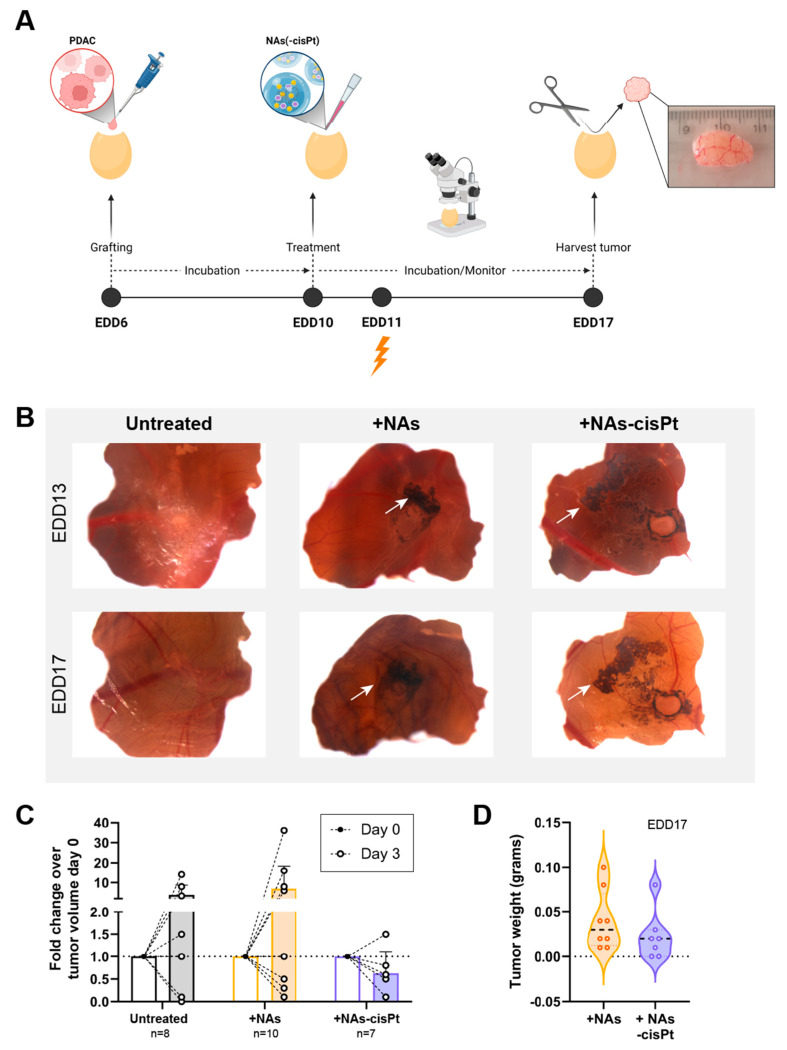
NAs-cisPt treatment in tumor-grafted PDAC3 on the CAM leads to tumor growth delay. (**A**) Schematic overview of the CAM assay and treatment. Created with BioRender. (**B**) Representative images of PDAC3-grafted tumors on the CAM at EDD13 and 17. White arrows indicate the presence of black aggregates surrounding the grafted tumor corresponding to the gold USNPs in both NAs and NAS-cisPt. Original magnification 20×. (**C**) The tumor volume on EDD10 and 13 and (**D**) tumor weight after harvest on EDD17 after treatment with NAs (total of 16.8 μg gold USNPs per egg administered) or NAs-cisPt (213 μM cisPt prodrug, 16.8 μg gold USNPs per egg administered) for 1 week on tumor-grafted PDAC3 on the CAM.

As the cisplatin prodrug and gold USNPs are encapsulated, the chorioallantoic membrane (CAM) in vivo model was used to investigate the effect of NAs-cisPt after uptake into the tumor using PDAC3 and SUIT2-028. Previously, we established PDAC3 cells to have good tumor-grafting rate, while retaining all the histopathological and genetic characteristics [22]. Tumor-bearing eggs were randomized into different treatment groups, monitored and harvested for further analysis (Figure 4A). The data showed that one week of treatment with 213 μM active cisplatin and 16.8 μg gold USNPs in NAs-cisPt per egg did not negatively affect the survival of chick embryos (seven out of seven eggs survived). Topical application of NAs(-cisPt) showed black aggregates on top of the CAM and surrounding the tumor, as depicted by the white arrows (Figure 4B). Detection via ICP-MS demonstrated that harvested tumors had an average ± S.D. of 9.86 ± 6.74% Au, 0.11 ± 0.19% Pt and 1.74 ± 1.84% Au, 2.28 ± 2.7% Pt of administered dose in CAM-PDAC3 after NAs and NAs-cisPt treatment, respectively (Supplemental Figure S3). Similar to NAs-cisPt-treated CAM-derived PDAC3, tumors were found to have taken up 1.06 ± 1.05% Au and 2.58 ± 3.15% Pt of the administered dose. Thus, platinum seems to have a more pronounced long-term persistence in tumors (probably due to DNA adduct formation) with respect to the gold USNPs as the gold-to-platinum ratio in NAs-cisPt is fixed to 6.1 ± 2.1. Fold change in tumor volume after 3 days of treatment shows an initial decrease for NAs-cisPt-treated samples compared to untreated and NAs-treated samples (Figure 4C), whereas harvested tumors at the end of the experiment were close in weight (Figure 4D, Supplemental Figure S4), suggesting that NAs-cisPt treatment results in delayed tumor growth in CAM-derived PDAC3 tumors.

**Figure 5:**
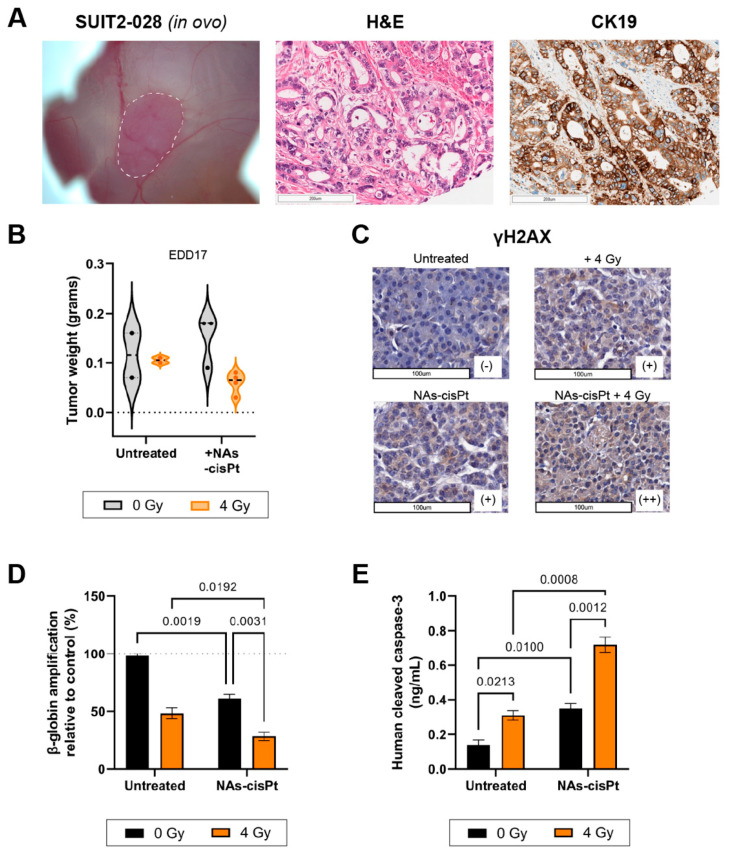
NAs-cisPt treatment combined with ionizing radiation enhances DNA damage and apoptosis induction in SUIT2-028 in ovo model. (**A**) Images of SUIT2-028-formed tumor on the CAM delineated in white dashed line (left), H&E (middle) and CK19 (right) staining from harvested SUIT2-028-grafted CAM tumors on EDD17. Scale bar represents 200 μm. (**B**) The tumor weight after harvest on EDD17 after NAs-cisPt (213 μM cisPt prodrug, 16.8 μg gold USNPs) treatment with or without 4 Gy radiation for 1 week on tumor-grafted SUIT2-028 on the CAM. Induction of DNA damage response was assessed through (**C**) γH2AX staining of CAM-derived SUIT2-028 tumors. Scale bar represents 100 μm. Scoring was performed by pathologists. (**D**) Assessment of β-globin amplifications via XL-PCR from SUIT2-028 (CAM) tumors after single or combination treatment. (**E**) Cleaved caspase-3 levels were assessed from CAM-derived SUIT2-028 tumors. Data represent the mean ± S.D. from 2 to 3 individual tumors in panel B, two individual experiments performed in duplicates in panel C and two biological experiments in duplicates.

The PDAC cell line SUIT2-028 had not been grafted onto the CAM before; therefore, we first investigated the histopathological characteristics of the tumor. Hematoxylin and eosin (H&E) staining of harvested SUIT2-028 CAM-derived tumors showed typical ductal structures that are associated with PDAC with stroma surrounding the ducts in between, whereas cytokeratin 19 (CK19) confirmed the presence of PDAC cells aligning the ducts, as previously reported [22,23] (Figure 5A). However, the majority of chick embryos grafted with SUIT2-028 did not survive the end of the experiment due to excessive tumor growth resulting in early death, whereas chick embryos treated with cisplatin or NAs-cisPt survived until time of harvest, leading to a diminished number of surviving eggs at the end of the experiment. Tumor weight after treatment with NAs-cisPt did not differ with the untreated group, but the addition of 4 Gy IR reduced the weight for both untreated and NAs-cisPt conditions (Figure 5B).

In a similar manner to our in vitro results, γH2AX staining showed increased DNA damage after one week of treatment with NAs-cisPt and further enhanced staining upon combination with 4 Gy. Beta-globin amplifications also confirmed the accumulation of DNA damage when SUIT2-028 CAM-tumors were treated with both NAs-cisPt and IR (Figure 5D). Lastly, cleaved caspase-3 levels were significantly increased in SUIT2-028 CAM-tumors treated with combined NAs-cisPt and 4 Gy radiation, resulting in apoptosis induction (Figure 5E).

## 4. Discussion

While the therapeutic efficacy of Pt(IV), derivatives of platinum compounds have been widely studied as a single agent [24,25,26], studies pertaining to their effect in combination with radiation are limited. In this study, we assessed the therapeutic efficacy of combination treatment of encapsulated a cisplatin Pt(IV) derivative and 3 nm gold USNPs with IR. Our findings show that the cisplatin prodrug in NAs-cisPt could delay the DNA damage response (DDR) induced by IR, as a result giving rise to the induction of apoptosis. This report highlights the feasibility of combining IR with cisplatin Pt(IV) derivatives through exploitation of nanotechnology.

The therapeutic efficacy of NAs-cisPt and IR was investigated in both in vitro and in ovo models. Combined results from DNA and protein levels as determined by XL-PCR and Western blots demonstrate an increase in DNA damage and delayed onset of DDR following NAs-cisPt treatment in combination with IR as shown via γH2AX. Expression of phosphorylated H2AX at position Ser139 peaked after 30 min up to 2 h of IR-induced DNA damage and subsequently diminished after 6 h in both SUIT2-028 and PDAC3 cells, consistent with previous studies in other tumor cell lines [27,28]. Despite being a characteristic marker for the induction of DNA double-stranded breaks and DDR, cisplatin has been shown to cause upregulation of γH2AX [27,29,30]. As expected, equal concentration of active cisplatin prodrug of NAs-cisPt showed increased γH2AX expression up to 24 h post radiation comparable to free cisplatin, indicating a similar effect takes place with the release of the cisplatin prodrug to be the cause. This is speculated to be the indirect cause of interstrand cross-links (ICLs), one of the common DNA damage products generated by platinum compounds, leading to stalled replication forks with auto-phosphorylated ATM to phosphorylate downstream H2AX [5,31].

Although a few studies have investigated the effect of gold USNPs in combination with radiation, it remains difficult to compare the results as size, shape, surface charge and coating can influence the effect in biological systems [32,33]. Therefore, we also investigated the effect of our gold USNPs at a diameter of 3 nm with and without radiation. Results from our cell viability assays showed increased inhibited proliferation when cells were treated with a higher gold USNP concentration present in NAs-cisPt. These results suggest a cytotoxic effect of gold USNPs in PDAC cells, as reported before [13]. However, contrary to our results from the cell viability assays, combination with IR and NAs did not affect the γH2AX expression, which can be explained due to a lower amount of gold USNPs used during NAs treatment in order to correlate to the same amount of gold USNPs present in NAs-cisPt. This correlates to decreased apoptosis induction in our in vitro results. Additionally, NAs-treatment of CAM-derived PDAC3 tumors did not alter the tumor volume nor tumor weight compared to NAs-cisPt. Taken together, the cytotoxic effect and subsequent apoptosis induction can be mostly ascribed to the cisplatin Pt(IV) prodrug component within NAs-cisPt. Albeit 3 nm gold USNPs exhibit cytotoxic properties in vitro at a 1.8-fold higher concentration, our findings do not demonstrate radiosensitization with the current conditions in our PDAC models. This correlates to ICP-MS data as reflected by a relatively low presence of gold and platinum found in our CAM models based on administrated total dose. However, even as a smaller fraction of gold, but in particular platinum, reaches the tumor, increased DNA damage and induction of apoptosis induced by IR preceding NAs-cisPt treatment demonstrates the feasibility of this combined treatment.

Although cisplatin is not part of the conventional treatment against PDAC, combinations of cisplatin with other chemotherapeutics as polychemotherapeutic regimens were increasingly studied in phase I-III trials [34,35,36,37]. From these multicenter trials, polychemotherapeutic strategies yield better outcomes in progression-free survival (PFS) and overall survival, as reflected in the existing multidrug regimen FOLFIRINOX as first-line treatment for locally advanced and metastatic PDAC patients [38,39]. Unfortunately, increased incidence of severe side effects restricts four-drug combinations in their clinical efficacy. Based on previous studies of polychemotherapeutic regimens using cisplatin-based multidrug regimens, polymorphism of the *Xeroderma pigmentosum group D* (*XPD*) correlates to increased risk of death and progression [40,41]. In particular, PDAC patients harboring the genotypes *XPD Gln751Gln* correlated with a worse prognosis and shorter PFS compared to patients with *XPD Lys751Lys* and *Lys751Gln*, but only for cisplatin-based treatments. The XPD protein acts as a helicase to unwind the DNA for DNA repair as part of the nucleotide excision repair (NER) pathway to resolve platinum adducts and intrastrand cross-links [42]. The existence of polymorphic *XPD* might also explain the differences seen in our cell viability results after treatment with cisplatin and NAs-cisPt in our PDAC models. Primary PDAC3 was inherently more resistant to both treatments as compared to PDAC cell lines MIA PaCa2 and SUIT2-028, as reflected by the resulting IC_50_ concentrations for both treatments. This is further reflected from the observed tumor weight from PDAC3 and SUIT2-028-derived CAM tumors. Although the sample size was considerably smaller, SUIT2-028-derived CAM tumors did appear to trend towards a smaller tumor after NAs-cisPt treatment. From our own data, genotyping revealed MIA PaCa2 to be heterozygous while PDAC3 is homozygous for the Gln751 allele. As implicated by previous studies, the Lys751 allele could have implications in decreased DNA repair efficiency via the NER pathway and mechanistically as a consequence of instable mRNA products [43,44]. Therefore, genotyping might help to select patients based on *XPD* who benefit from cisplatin-based treatments.

While co-treatment of PDAC with NAs-cisPt and IR shows promising results, further investigations are needed to optimize the concentration of gold USNPs in the NAs. Of note, GNP-associated toxicity has been reported previously in both normal and tumor cells, as they can be chemically reactive to other vital cellular structures, including the membrane and mitochondria and accumulate in organs such as the spleen, liver and kidney in rats [45,46,47]. While our gold USNP-loaded NAs did not induce significant alterations in reproductive functions nor toxic side effects in zebrafish larvae, further investigation and monitoring for long-term periods are of importance, in particular when combining with IR [32]. Furthermore, the choice to encapsulate gold USNPs instead of using itself as a vehicle such as our NAs, could improve unspecific binding in various tissues.

Of note, the delivery of chemotherapeutic agents to PDAC remains a big challenge due to poor vascularization and dense stroma. While the “enhanced permeability and retention” (EPR) effect might not be an optimal strategy to delivery nanomaterials to PDAC, the nano-architectures presented in this study are a step forward to the potential treatment of this neoplasm as their surface can be functionalized with molecules that may selectively potential biomarkers highly expressed in PDAC cells, providing additional specificity and efficacy. This is exemplified by transferrin-conjugated NAs in 3D spheroid models of PDAC cells as NAs were found to be significantly internalized [48]. Importantly, the emergence and improved clinical efficacy proven with existing nanomedicine in PDAC treatment such as albumin-bound paclitaxel (also shortened for nab-paclitaxel) and liposomal irinotecan further encourages the application of nanocarriers [49,50].

An important limitation of the present study is that the in ovo models cannot reproduce the dense stromal reaction of PDAC [51]. Moreover, this is a very ischemic tumor while the CAM is well vascularized. However, the CAM tumor models take advantage of innate physiological features of oxygen and nutrients flux that are hardly replicated in co-culture systems or organoids and provide practical and technical ease compared to murine models [19]. Meanwhile, it would be interesting and worthwhile to perform future experiments of co-cultures of pancreatic cancer and stellate cells engrafted on CAM.

However, further studies on murine models with intraperitoneal administration would provide additional information about the pharmacokinetics and if the encapsulated cisplatin pro-drug and gold nanoparticles are selectively released in the tumor, as described in a recent study on mesoporous silica nanoparticle-based platform for the target-specific, spatiotemporal, ratiometric, and safe co-delivery of gemcitabine and cisplatin [52].

Lastly, acquired resistance against cisplatin is multifactorial as demonstrated by Mecenzev et al., which is why further investigations are required to identify the underlying resistance mechanisms against cisplatin or oxaliplatin (as part of FOLFIRINOX regimen or as gemcitabine–cisplatin combination, that is used in several patients because it is less toxic than the FOLFIRINOX scheme) to further select patients for personal precision medicine [53]. Of note, this approach could also provide new tools for neoadjuvant chemotherapy, which is emerging as a potential strategy for the treatment of PDAC patients [54].

## 5. Conclusions

To summarize, we assessed the therapeutic efficacy and DDR of combined treatment of cisplatin Pt(IV) prodrug-loaded NAs and radiation in pancreatic cancer models. We showed that preceding treatment with NAs-cisPt could enhance IR-induced DNA damage leading to increased apoptosis. These findings highlight the feasibility and potential of encapsulated chemotherapeutics and radiosensitizing agents as a platform to further improve precision nanomedicine and encourage the development of multimodality treatment.

## Figures and Tables

**Table 1:** Overview of IC_50_ concentrations of cisplatin and NAs-cisPt (41.92 μg/mL) as determined by SRB after 72 h of treatment in PDAC cultures MIA PaCa2, SUIT2-028 and PDAC3. Data show the mean ± S.E.M. from three individual experiments in triplicate.

PDAC Cells	IC_50_ Cisplatin (μM)	IC_50_ NAs-cisPt (μM)
MIA PaCa2	8.43 ± 1.89	1.16 ± 0.41
SUIT2-028	1.28 ± 0.08	0.78 ± 0.08
PDAC3	11.48 ± 3.02	>50

## Data Availability

The data presented in this study are available in this article.

## Supplementary Materials

The following supporting information can be downloaded at: https://www.mdpi.com/article/10.3390/cancers14123034/s1, File S1: Synthesis and preparation of cisplatin prodrug-loaded ultrasmall-in nano architectures; Figure S1: Cell viability of MIA PaCa2 cells after 72 h of treatment with increasing concentrations of NAs-cisPt pre-loaded with different starting amounts of gold USNPs; Figure S2: Uncut, original Western blots used to assemble Figure 3 in the manuscript; Figure S3: Distribution plots of gold and platinum detected via ICP-MS in CAM-derived PDAC3 and SUIT2-028 tumors; Figure S4: Distribution plots of the tumor volume in mm^3^ of grafted PDAC3 tumors on the CAM.
