# Supplementary material for: Biodegradable Ultrasmall-in-Nano Architectures Loaded with Cisplatin Prodrug in Combination with Ionizing Radiation Induces DNA Damage and Apoptosis in Pancreatic Ductal Adenocarcinoma
Source: Cancers (Basel). 2022 Jun 20;14(12):3034. doi: 10.3390/cancers14123034 (PMC9221262; doi:10.3390/cancers14123034)
Supplement: Supplementary file 1 [file cancers-14-03034-s001.zip › Supplemental Figures S1-S4.pdf]

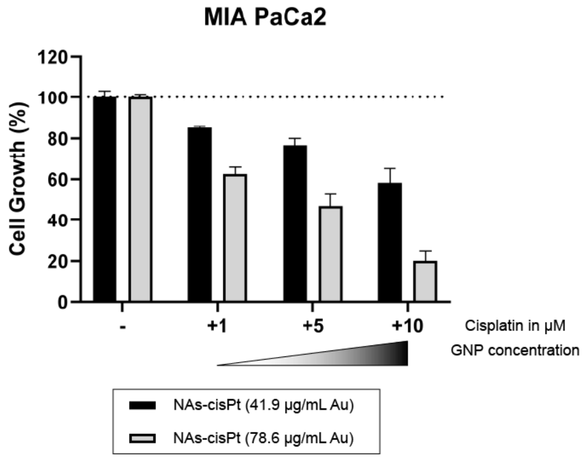

Supplemental Figure S1. Cell viability of MIA PaCa2 cells after 72 hours of treatment with increasing concen-trations of NAs-cisPt pre-loaded with different starting amounts of gold USNPs. As such, the total number of gold USNPs for each concentration of cisplatin increases as represented by the triangle bar. Data represents the mean ± S.D. from one individual experiment performed in triplicates. Abbreviations: Gold USNPs = gold ultrasmall nanoparticles.

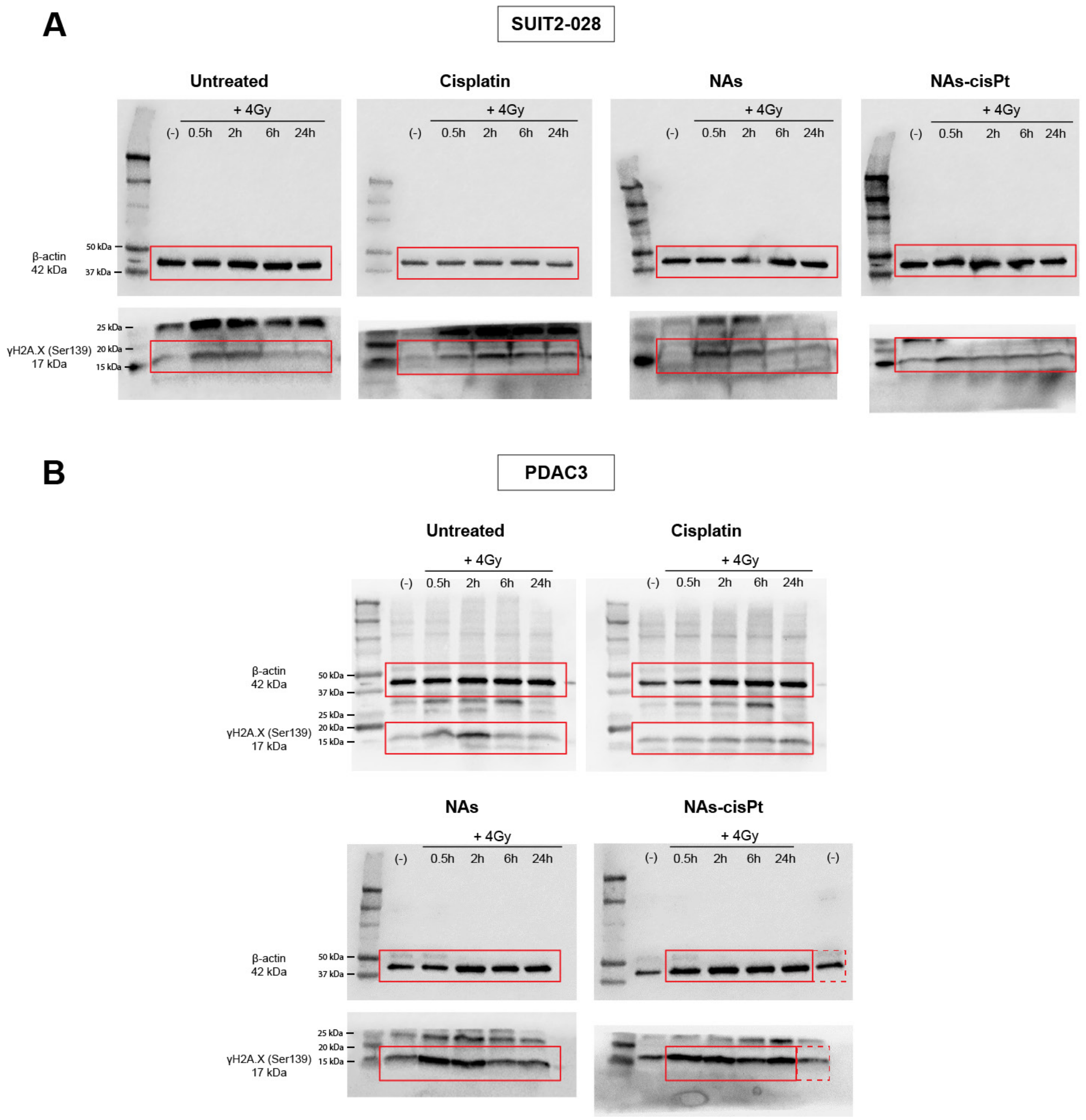

Supplemental Figure S2. Uncut, original Western blots used to assemble Figure 3 in the manuscript. (**A**) SUIT2-028 and (**B**) PDAC3 after single drug or combined with radiation treatment. Cells were treated with 1 µM cis-platin, NAs and 1 µM NAs-cisPt for 24 hours followed with or without 4 Gy radiation. The unirradiated (-) condi-tion was collected simultaneously as the 0.5h post-irradiation sample. NAs and NAs-cisPt corresponded in the same amount of gold USNPs (µg Au). The molecular weights of phosphorylated γH2AX (Ser139) and β-actin correspond to 17 kDa and 42 kDa, respectively. Red boxes represent the area of the blots cut for Figure 3A. The band left and right of the red box in PDAC3 — NAs-cisPt corresponds to the unirradiated condition, with the left sample loaded at an incorrect volume. Dashed, lined, red box of NAs-cisPt-treated PDAC3 cells with-out 4 Gy got re-arranged for Figure 3A, with the correct volume totalling 50 µg of total protein. All conditions are loaded to a total of 50 µg of proteins. Additional bands in the γH2AX blots at 25 kDa correspond to mono-ubiq-uitinated γH2AX. Abbreviations: Gold USNPs = gold ultrasmall nanoparticles; NAs = nanoarchitectures.

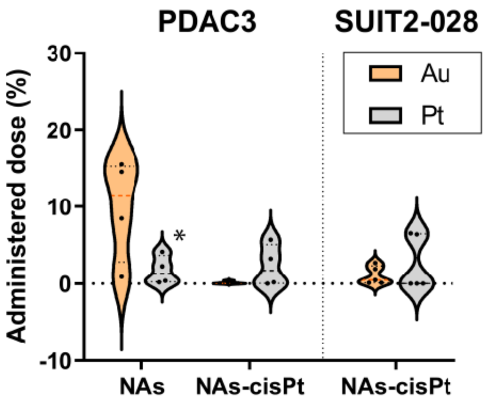

Supplemental Figure S3. Distribution plots of gold and platinum detected via ICP-MS in CAM-derived PDAC3 and SUIT2-028 tumors. Gold (Au) and platinum (Pt) from administered NAs and NAs-cisPt were plotted against the total ad-ministered dose. Total administered dose corresponded with 16.8 µg Au, and 16.8 µg Au and 2.06 µg Pt in NAs and NAs-cisPt, respectively. Abbreviations: CAM = chorioallantoic membrane; ICP-MS = inductively coupled plasma - mass spectrometry; NAs = nanoarchitectures. * Detected Pt in NAs <0.01 µg Au.

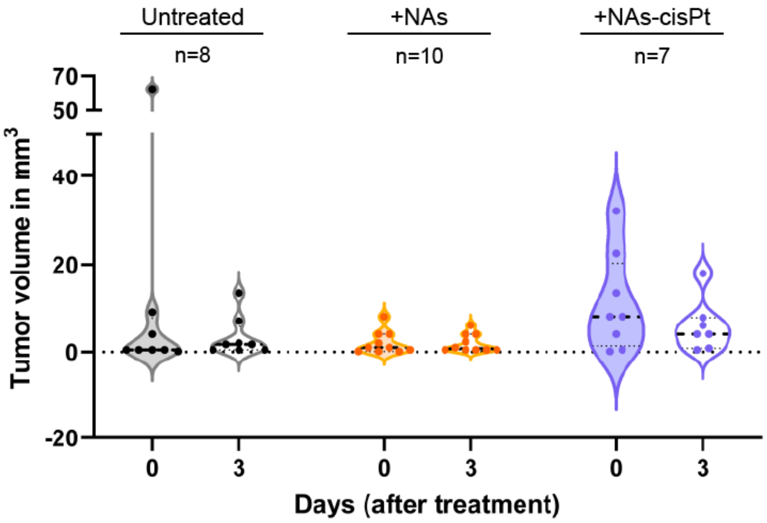

Supplemental Figure S4. Distribution plots of the tumor volume in mm^3 of grafted PDAC3 tumors on the CAM. Tumor volume was measured on EDD10 - corresponding to day 0 - and EDD13. Abbreviations: CAM = cho-rioallantoic membrane; EDD = embryonic developmental day; NAs = nanoarchitectures.
