# Supplementary material for: Biodegradable Ultrasmall-in-Nano Architectures Loaded with Cisplatin Prodrug in Combination with Ionizing Radiation Induces DNA Damage and Apoptosis in Pancreatic Ductal Adenocarcinoma
Source: Cancers (Basel). 2022 Jun 20;14(12):3034. doi: 10.3390/cancers14123034 (PMC9221262; doi:10.3390/cancers14123034)
Supplement: Supplementary file 1 [file cancers-14-03034-s001.zip › Supplemental File S1.pdf]

**Supplemental File S1: Synthesis and preparation of cisplatin prodrug-loaded ultrasmall-in nano architectures**

*Preparation of Cisplatin Prodrug-Modified Poly(L-Lysine)*

The cisplatin prodrug c,t,c-$[PtCl_2(NH_3)_2(OH)(O_2CCH_2CH_2CO_2H)]$ was synthesized as specified elsewhere [1,2]. To covalently link to poly(L-lysine), 12 mg of the prodrug was dissolved in 100 µL of phosphate-buffered saline (PBS) prepared at pH 7.4. The prodrug solution was added with a freshly prepared mixture of 1-Ethyl-3-(3-dimethylaminopropyl)carbodiimide (EDC) and n-Hydroxysuccinimide (NHS) that were also dissolved in 100 µL PBS together (25 mg EDC/15 mg NHS). After 20 minutes of mixing, the prodrug–EDC/NHS mixture was added with 750 µL of aqueous solution of poly(L-lysine) (PL; 15-30 kDa; 30 mg), and the resulting mixture was incubated while shaking (700 rpm) overnight at room temperature. The product (PL-cisPt) was recovered using Amicon 10 kDa centrifugal filter, in which the residue was washed thrice with milliQ water via centrifugation. After the final washing, the product was resuspended in 1 mL milliQ water and was stored at -20°C until use.

*Synthesis of Gold Ultrasmall Nanoparticles and Polymeric Arrays*

In 20 mL of milliQ water, 200 µL of tetrachloroauric (III) acid ($HAuCl_4$; Alfa Aesar, ACS 99.99% metal basis; stock: 10 mg/mL in milliQ water) was mixed with 10 µL poly(sodium 4-styrene sulfonate) (PSS; 70 kDa; 30% aqueous solution). Gold USNPs were prepared through reduction of the gold ions after a quick addition of 200 µL of sodium borohydride (stock: 8 mg/mL in milliQ water) on a vigorously stirring solution. The solution was aged for another 10 minutes. Then, 75 µL of PL (stock: 40 mg/mL in milliQ water) for standard NAs or 165 µL of PL-cisPt for NAs-cisPt was added to the gold USNPs suspension and was incubated for another 20 minutes. Finally, the gold USNP polymeric arrays were collected by centrifugation at 16873 x g for 5 minutes and were then resuspended in 2 mL of milliQ water.

*Synthesis of Nano-Architectures*

A modified Stöber process was followed to grow a silica shell on the periphery of gold USNPs' polymeric arrays. Two 50 mL tubes were filled each with 35 mL ethanol, 1.2 mL ammonia solution (Merck, 32%), 20 µL tetraethyl orthosilicate, and 1 mL Au polymeric array. The solutions were incubated with moderate shaking for 3 h. Then, resulting nano-architectures (NAs or NAs-cisPt) were collected after a 30-minute centrifugation at 3220 x g. The supernatant was discarded, and the products were resuspended in and washed with ethanol. After centrifugation at 14000 rpm for 3 minutes, the ethanol supernatant was discarded, and the nano-architectures were again resuspended in ethanol for another wash cycle. After discarding the second washing supernatant, the nano-architectures were again resuspended in ethanol and then spun for 15 seconds or until the rotational speed reached 14000 rpm. This step removed the larger nano-architectures and retained those that were approximately 150-200 nm in diameter. After separating the supernatant, the smaller nano-architectures were again collected after centrifugation at 14000 rpm for 3 minutes. The ethanol supernatant was discarded and the final nano-architectures were resuspended and stored in 1 mL ethanol and at -20°C, as described previously [3].

## References


1. Cassano, D.; Santi, M.; Cappello, V.; Luin, S.; Signore, G.; Voliani, V. Biodegradable Passion Fruit-Like Nano-Architectures as Carriers for Cisplatin Prodrug. *Particle & Particle Systems Characterization* **2016**, *33*, 818–824, doi:10.1002/ppsc.201600175.
2. Dhar, S.; Daniel, W.L.; Giljohann, D.A.; Mirkin, C.A.; Lippard, S.J. Polyvalent Oligonucleotide Gold Nanoparticle Conjugates as Delivery Vehicles for Platinum(IV) Warheads. *J. Am. Chem. Soc.* **2009**, *131*, 14652–14653, doi:10.1021/ja9071282.
3. Sarogni, P.; Mapanao, A.K.; Gonnelli, A.; Ermini, M.L.; Marchetti, S.; Kusmic, C.; Paiar, F.; Voliani, V. Chorioallantoic Membrane Tumor Models Highlight the Effects of Cisplatin Compounds in Oral Carcinoma Treatment. *iScience* **2022**, *25*, 103980, doi:10.1016/j.isci.2022.103980.
